# Converging and Diverging Cerebellar Pathways for Motor and Social Behaviors in Mice

**DOI:** 10.1007/s12311-024-01706-w

**Published:** 2024-05-23

**Authors:** Meike E van der Heijden

**Affiliations:** 1 Fralin Biomedical Research Institute, Virginia Tech Carilion, Roanoke, VA USA; 2 Center for Neurobiology Research, Virginia Tech Carilion, Roanoke, VA USA; 3 School of Neuroscience, Virginia Tech, Blacksburg, VA USA

**Keywords:** Cerebellum, Motor control, Social behaviors, Mice, Circuits, Genetic models

## Abstract

Evidence from clinical and preclinical studies has shown that the cerebellum contributes to cognitive functions, including social behaviors. Now that the cerebellum’s role in a wider range of behaviors has been confirmed, the question arises whether the cerebellum contributes to social behaviors via the same mechanisms with which it modulates movements. This review seeks to answer whether the cerebellum guides motor and social behaviors through identical pathways. It focuses on studies in which cerebellar cells, synapses, or genes are manipulated in a cell-type specific manner followed by testing of the effects on social and motor behaviors. These studies show that both anatomically restricted and cerebellar cortex-wide manipulations can lead to social impairments without abnormal motor control, and vice versa. These studies suggest that the cerebellum employs different cellular, synaptic, and molecular pathways for social and motor behaviors. Future studies warrant a focus on the diverging mechanisms by which the cerebellum contributes to a wide range of neural functions.

## Introduction

Reports of cerebellar malformations and lower Purkinje cell numbers in patients with autism spectrum disorder (ASD) date back to the 1980s [1–3]. Since then, findings of cerebellar pathologies have been consistent in patients with ASD [4–7]. These cerebellar abnormalities may explain the high prevalence of disturbances in motor coordination, one of the cerebellum’s most well-characterized functions, in infants with ASD [8–10]. More recently, there has been a consensus that the cerebellum also directly contributes to neural functions that are more prominently associated with ASD, including social cognition [11, 12]. Indeed, anatomical, genetic, developmental, and functional studies in patients all point towards a central role for the cerebellum in ASD pathophysiology [13–18]. However, studies in patients cannot exclude the possibility that dysfunction in other brain regions has confounded findings of the cerebellum’s contribution to social deficits in ASD.

In the early 2010s, a study was published in which the ASD-associated gene *Tsc1* was deleted solely from cerebellar Purkinje cells in mice. These mice displayed abnormal motor control and social deficits, thereby providing the first direct evidence that cerebellum-restricted manipulations can cause motor and social impairments [19]. Numerous other papers published in the last decade have confirmed that the cerebellum contributes to social behaviors in mice and that cerebellar dysfunction directly contributes to deficits observed in patients with ASD [20–22].

With the consensus that the cerebellum contributes to a broader range of behaviors than exclusively motor control and that cerebellar dysfunction underlies more disorders than just movement disorders, a new question arises: does the cerebellum contribute to social behaviors in a similar manner as it does to motor control? Cerebellar input and output pathways have a high degree of functional segregation [23–31], yet the cerebellar cortex consists of highly repetitive microcircuits that exhibit relatively few regional differences [32, 33]. The repetitive nature of cerebellar microcircuits has led to the theory that the cerebellum may mediate many different behaviors through a similar neural mechanism [34]. According to this theory, diverse cerebellar functions are mediated by selective inputs and outputs to anatomically and functionally distinct brain regions but through similar computations within the cerebellar cortex. There is some debate about the validity of this model [35], and additional theories of cerebellar function have been proposed [36, 37], but no direct evidence in favor or against a singular cerebellar function has been provided to date.

If all cerebellar functions are mediated through a similar circuit or cellular pathways, one would hypothesize that discordance between motor and social impairments could only be derived from anatomically constrained manipulations of the cerebellar cortical neurons or by selective manipulation of cerebellar input or outputs. This review sets out to find whether mouse studies support or contradict this hypothesis. The review will focus on experimental mouse models wherein the cerebellar contributions to motor control and social behaviors are investigated using cerebellum-specific manipulations to avoid confounding by other brain regions. Before summarizing the mouse behavioral studies, the review will introduce the anatomical structure of the mouse cerebellum, and the framework of a similar neural mechanism for all cerebellum-dependent behaviors is further delineated.

### The (Non)Uniformity of Cerebellar Anatomy

**Fig. 1:**
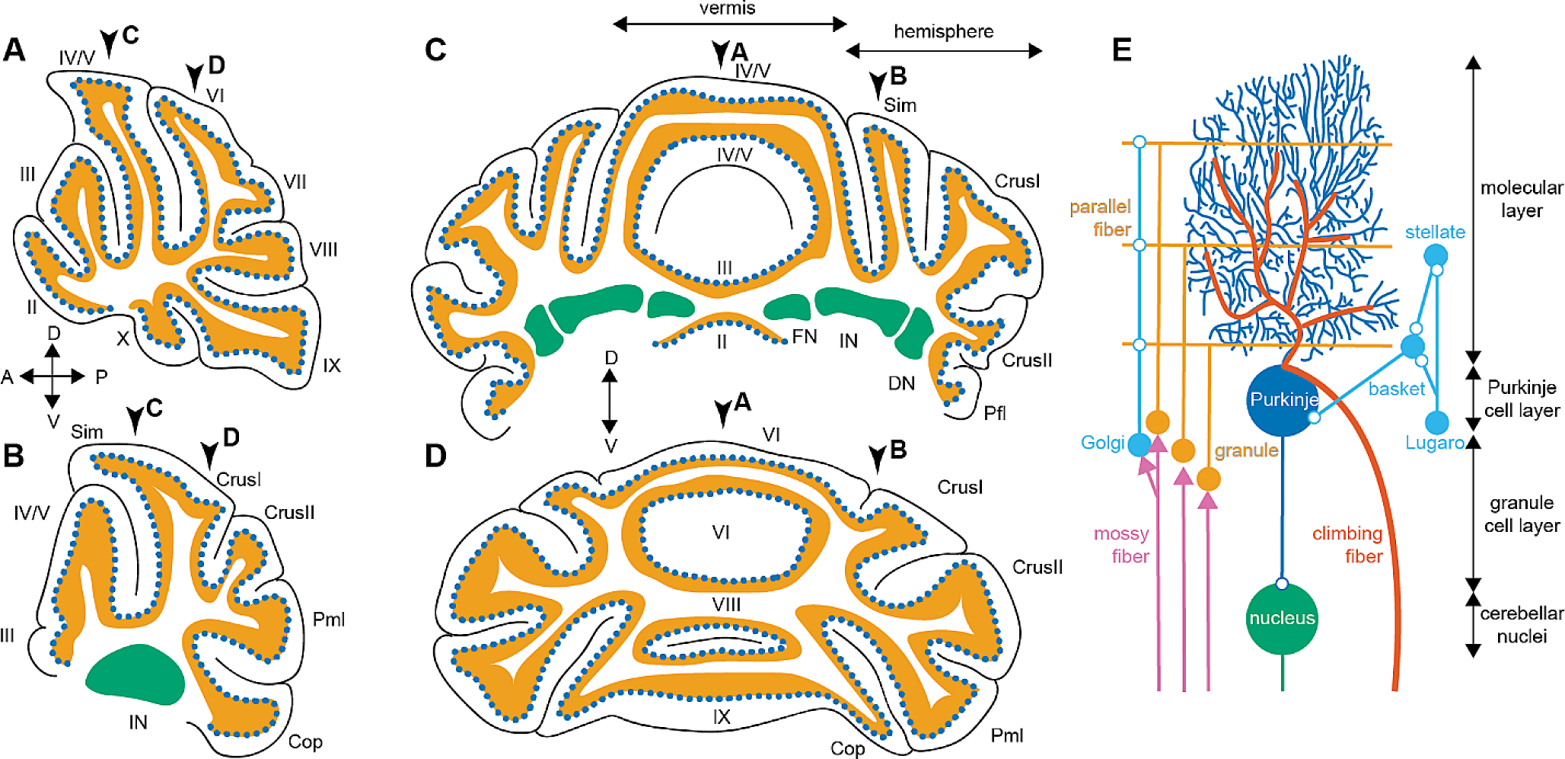
Overview of cerebellar anatomy and circuit. **(A)** Schematic of the lobular organization of the vermis in the sagittal plane. **(B)** Schematic of the lobular organization of the hemisphere in the sagittal plane. **(C)** Schematic of the lobular organization of the anterior cerebellum in the coronal plane. **(D)** Schematic of the lobular organization of the posterior cerebellum in the coronal plane. For **A – D**: granule cell layer in orange, Purkinje cell layer in dotted blue, and cerebellar nuclei in green; arrows point to the approximate coordinates of the other cross sections in **A – D**. **(E)** Schematic of the cellular architecture of cerebellar microcircuits. Only cell types and connectivity that are universally present across the cerebellum are depicted in this summary schematic. Open round circles are inhibitory synapses, arrowheads are excitatory synapses, and parallel and climbing fibers are also excitatory. *Abbreviations* Sim = lobule simplex, Pml = paramedian lobule, Cop = copula pyramidis, FN = fastigial nucleus, IN = interposed nucleus, DN = dentate nucleus, A = anterior, P = posterior, D = dorsal, V = ventral

Compared to other brain regions, specifically the cerebral cortex, the cerebellum has a surprisingly homogeneous cellular architecture across its cortex – which has led to hypotheses that the cerebellum modulates different behaviors through similar cellular and synaptic pathways. The cerebellum is the only foliated structure in the mouse brain, which allows for anatomical compartmentalization across rostrocaudal and mediolateral folds (Fig. 1). These anatomical regions are partially aligned with certain cerebellar-dependent behaviors [38] but results from human studies put into question whether functional boundaries fully follow anatomical regions [39–41].

The cerebellum’s connectivity to brain regions involved in social and motor behaviors is regulated through a multi-synaptic bi-directional circuitry. It receives information from the forebrain and motor cortices via the pontine nuclei. There is also evidence that these brain regions may communicate with the cerebellar cortex via climbing fibers originating in the inferior olive. The cerebellum, in turn, communicates its signals to the forebrain and sensory-motor cortices through di-synaptic pathways via the thalamus. This circuitry results in potentially parallel closed-loop circuits that may mediate diverse behavioral domains [27, 30, 42–44].

Much of the functional specification of anatomical regions is thought to arise from task-specific information entering the cerebellum through two types of glutamatergic afferents: mossy fibers and climbing fibers. Mossy fibers relay spinal, vestibular, or cerebral-cortical information to the cerebellum in diverse anatomical regions (spinal: lobules VIII and IX; vestibular: lobules IX and X; cerebral-cortical: lobules VI, VII, and VIII) [45–47]. Climbing fibers originate in the inferior olive, and their inputs from the cerebral cortex [48] and outputs towards the cerebellar cortex are also anatomically organized [30, 49].

Most information leaving the cerebellum is carried through cerebellar nuclei neurons in the fastigial, interposed, and dentate nuclei. These nuclei are composed of intermingled glutamatergic, GABAergic, and glycinergic neurons. Nuclei neurons receive input from neurons in the same mediolateral plane; therefore, nuclei neurons receive indirect, processed information from specific modules of mossy and climbing fiber afferents [28, 30, 50, 51]. However, information from multiple posterior-anterior lobules can also converge onto single nuclei neurons, suggesting that multi-model nuclei neurons may exist [52]. Nuclei neurons send projections to many extra-cerebellar regions, including the midbrain, brainstem, and spinal cord [53], and single classes of nuclei neurons often send information to multiple downstream brain regions [29, 30, 54]. Most brain regions that receive information from glutamatergic nuclei neurons also receive inputs from the GABAergic [29] or glycinergic nuclei neurons [55], suggesting some parallel processing by cerebellar nuclei neurons with different neurotransmitter identities. Therefore, like cerebellar inputs, cerebellar outputs may have different functions based on their connectivity patterns.

In contrast to the region-specific input pathways and the variety of cerebellar output pathways, the local architecture in the cerebellar cortex is highly stereotyped across cerebellar lobules. Mossy fibers terminate onto granule cells which in turn make connections onto Purkinje cells via parallel fibers [56]. Purkinje cells also receive direct input via climbing fibers [57]. The cerebellar cortex is further composed of myriad GABAergic and glycinergic interneurons that are mostly uniform across the cerebellar cortex [58, 59]. Purkinje cells form the sole output of the cerebellar cortex, meaning that all cerebellar cortical computations involve these neurons. The cerebellar circuitry, depicted in Fig. 1, has some heterogeneity across different anatomical regions. For example, Purkinje cells receive feedback from nuclei neurons via granule cells [60, 61], directly inhibit granule cells [62], and have different dendritic morphology and climbing fiber input organization [57] in different cerebellar regions. Yet the cerebellar cortex shows undeniably less anatomical heterogeneity than that is observed in the cerebral cortex [44, 63–66], and functional differences based on anatomical differences across cerebellar cortical microcircuits are currently not understood.

### The (Non)Uniformity of Cerebellar Function

The theory that the cerebellum may contribute to different behavioral domains through similar pathways is derived from the observation that the anatomical architecture is relatively uniform across the cerebellar cortex. This theory further underscores that the cerebellum optimizes neural functions for cognition, movement, and social behaviors, rather than generating these behaviors [34]. When the cerebellum is dysfunctional, one does not lose the ability to perform certain actions, but rather the ability to perform them well. For example, many patients with cerebellar diseases lose the ability to make smooth, voluntary movements and are diagnosed with ataxia, dysmetria, or tremor [67–69]. These motor symptoms are dissimilar from those observed in patients with damages to primary motor pathways, like strokes in the motor cortex or spinal cord lesions, who display an inability to move part of their bodies [70, 71]. Similarly, patients with cerebellar disease also display impairments in non-motor functional domains, including social and emotional processing, cognition, and language, rather than the loss of these functions that is observed in patients with lesions in cortical brain regions [67, 72–74]. From these observations, a hypothesis emerged that the cerebellum contributes to a wide range of behaviors by serving as an oscillation dampener that maintains function around a homeostatic baseline [67]. When this homeostatic function is disrupted, patients can display motor dysmetria (ataxic movements) and/or cognitive dysmetria (impaired cognition and affect) [67, 72–74].

Another theory is that the cerebellum works as an internal model that represents (or predicts) the external world in neural signals [75, 76]. These internal models allow for quick adjustments of behavior by comparing the intended action (communicated through a collateral efference copy signal) and the actual outcome of behavioral demands (communicated through sensory feedback). When these internal models do not perform optimally, adjustments to movements can become imprecise and only rely on sensory feedback which may result in tremors and ataxic motor trajectories. In non-motor domains, the lack of a functional internal model may explain the behavioral rigidity observed in many patients with ASD [77, 78].

The climbing fiber synapse from the inferior olive (Fig. 1E) is often assumed to be the anatomical substrate for a learning or error message in the internal models [75, 79]. However, recent studies have shown that other neurons in the cerebellum can also encode errors [79]. Furthermore, there is evidence that climbing fibers and granule cells (Fig. 1E) can encode reward prediction and expectation in addition to errors [80–82]. Therefore, there is still debate whether a singular cerebellar function exists, what this function may be, and what the neural substrates for the cerebellar computations may be. Further research is needed to answer many of these outstanding questions [35, 79, 83, 84].

The goal of this review is to summarize data from experimental mouse studies to investigate whether the cerebellar cortex uses identical cellular, synaptic, and molecular pathways to optimize performance in different behavioral domains, with a focus on broad motor and social tasks. If there is a singular universal function for the cerebellum across functional domains, then the cellular and molecular pathways employed to transform cerebellar inputs would be identical for all cerebellum-dependent behaviors. Behavior-specific modulation by the cerebellum could then be arranged via different anatomical regions with behavior-specific connectivity to the cerebral cortex. If this is true, the following hypotheses should be supported by experimental studies:

1. Manipulations to *restricted areas* of the cerebellar cortex can result in impairments in *one* behavioral domain while preserving others.
2. Manipulations to specific cerebellar nuclei neuron *output subtypes* can result in impairments in *one* behavioral domain while preserving others.
3. Manipulations to cerebellar microcircuits across the *entire* cerebellar cortex will result in impairments across *all* behavioral domains.

This review tests these three hypotheses by summarizing opto-, chemo-, and conditional genetic mouse models with cerebellum-specific manipulations that were tested for at least one behavior in the social domain and one behavior in the motor domain. Based on the outlined hypotheses, a discordance of impairments between social and motor behaviors would only be expected when the cerebellum was manipulated in an anatomically restricted manner or when only specific cerebellar output pathways were perturbed.

### Regional Manipulations to the Cerebellar Cortex

First, this review summarizes findings from opto-, chemo-, or conditional genetic mouse models wherein anatomically restricted populations of Purkinje cells were manipulated and social and motor behaviors were tested. As summarized in the section “The (non)uniformity of cerebellar anatomy,” the cerebellar cortex receives information from functionally distinct brain regions via anatomically segregated input pathways onto distinct cerebellar lobules (Fig. 1A-D). If the cerebellum mediates diverse sets of behaviors through functionally specific input pathways, one expects that local Purkinje cell manipulations could result in social deficits without motor impairments, and vice versa, because the Purkinje cells in those lobules only process information related to the specific behavioral domains. Box1 describes some of the most frequently used behavioral paradigms for assessing social behaviors and motor control in mice and Table 1 summarizes the findings described in this section.

**Table:** 

**Box 1. Summary of mouse behavioral paradigms.**
**Social behaviors**
*Three Chamber Sociability Test*: In this test, a mouse is placed in a box that is separated into three sections. The middle section is empty, and on either side is a section that holds either an inanimate object or a stranger mouse. The time spent exploring each side of the box is measured. Healthy control mice usually spend more time exploring the stranger mouse than the inanimate object. Mice with impaired sociability have no preference between the object and the stranger mouse.
*Three Chamber Social Novelty Test*: This test is similar to the sociability test, with the difference being that the inanimate object is replaced by a familiar mouse. A healthy control mouse spends more time with the stranger mouse than the familiar mouse. Mice with impaired social recognition have no preference between the familiar and the stranger mouse.
*Vocalizations in Social Isolation Paradigm*: In this test, young pups are separated from their mothers, and the number and duration of their calls for their mothers is measured. Mice with impaired social vocalizations make fewer calls for their mothers than healthy control mice.
**Motor behaviors**
*Rotarod*: In this test, a mouse is placed on an accelerating rod, and time until falling is measured. Mice with impaired motor control spend less time on the rod than healthy control mice.
*Gait*: In this test, mouse gait is visualized by either letting mice with painted paws walk over paper or by measuring step width and length digitally using cameras. Mice with impaired motor control often have wider and shorter steps than healthy control mice.
*Developmental motor reflexes*: To test negative geotaxis and righting reflexes, young pups are placed nose down on an incline or on their backs, respectively. The duration to turn upwards or on to their four paws is measured. Mice with impaired developmental motor reflexes take more time to turn than healthy control mice.

A study published by Badura and colleagues provides perhaps the most extensive investigation of the contribution of different cerebellar lobules to different mouse behaviors [85]. In this study, the authors inhibited Purkinje cells by activating local interneurons in the molecular layer (Fig. 1E), which was accomplished by expressing a receptor that is activated by synthetic drugs (chemogenetics). They found that inhibition of Purkinje cells in Crus I or II (Fig. 1A-D) in juvenile mice caused impaired social preference without changes in gait. Another study found that direct chemogenetic inhibition of Purkinje cells in the right Crus I resulted in impaired sociability and social recognition, without affecting motor performance on the accelerating rotarod [86]. Chemogenetic Purkinje cell inhibition in the posterior vermis also resulted in reduced sociability and social recognition without affecting motor performance on the accelerating rotarod [42]. Interestingly, chemogenetic Purkinje cell activation in Crus I, but not the posterior vermis (Fig. 1A-D), rescued abnormal social behaviors caused by conditional deletion of an ASD gene from Purkinje cells [42]. Neither chemogenetic Purkinje cell activation in neither Crus I nor in the posterior vermis rescued abnormal motor performance on the accelerating rotarod [42]. These data suggest that Crus I is more important for social behaviors than motor control. However, Badura and colleagues showed that inhibition of Purkinje cells via chemogenetic activation of inhibitory neurons in Crus I in adult mice caused a wider step width (gait abnormality) without changing sociability [85]. This latter finding suggests that the type and timing of the experimental perturbation may also influence the effect on behavioral deficits.

Badura and colleagues further reported that inhibition of Purkinje cells through chemogenetic activation of inhibitory neurons in lobule VII (Fig. 1A-D) in adult, but not juvenile, mice caused impaired social preference without gait changes [85]. Others also found that inhibiting Purkinje cells through chemogenetic or optogenetic activation of inhibitory interneurons in posterior lobules VI/VII (Fig. 1A-D) resulted in impaired performance of a delayed social recognition test without changes in motor performance on the accelerating rotarod [86]. A study by a third group found that optogenetic excitation or inhibition of vermal lobule VII Purkinje cells (Fig. 1A-D) resulted in increased or decreased aggressive behavior towards an intruder mouse, respectively [87]. This group further confirmed that neither optogenetic manipulation affected motor performance on the rotarod, and only inhibition of Purkinje cells in lobule VII (Fig. 1A-D) resulted in a reduced social approach [87]. Therefore, similar to Crus I, lobule VII may be more important for social behaviors than motor control and the timing of the manipulation may contribute to the expression of the phenotype.

Finally, the effects of regionally restricted manipulations in the vermis of anterior lobules IV/V (Fig. 1A-D) on social and motor behaviors were tested in several different manners. Chemogenetic activation of inhibitory interneurons, and thereby inhibition of Purkinje cells in this region, led to impaired performance of a delayed social recognition test without changes in motor performance on the accelerating rotarod [86]. However, lesioning or chemogenetic activation of Purkinje cells in anterior lobules IV/V (Fig. 1A-D) resulted in impaired sociability *and* impaired performance on the accelerating rotarod [88]. These results further underscore that it is not just which neurons are impaired but also how the neurons are impaired that influences the effect on behavioral deficits. Some studies further show that the timing of the perturbation may influence which behavioral domain was affected [85, 89]. Additional research is needed to understand why the nature and timing of cerebellar manipulation can influence how cerebellar-dependent behaviors are affected. Nevertheless, the cited studies underscore that manipulating anatomically restricted Purkinje cell populations can result in motor impairments without social impairments, and vice versa (Table 1).

### Manipulations to Cerebellar Aminergic Signaling

Two studies specifically investigated how cerebellar aminergic signaling in the cerebellar cortex influences cerebellum-dependent behaviors. The first study deleted tyrosine hydroxylase (TH), an enzyme essential for the synthesis of catecholamines, from all Purkinje cells [90]. TH is not universally expressed throughout the cerebellar cortex; rather, TH is present in a large proportion of Purkinje cells in lobules VII, VIII, and IX, and the flocculi (Fig. 1A-D), making this neither a strictly local nor global manipulation. Mice lacking TH in Purkinje cells had impaired social recognition memory but not motor impairments as tested on the accelerating rotarod [90].

The second study described the role of cerebellar dopamine D2 receptors in a multitude of cerebellar behaviors [91]. About half of all Purkinje cells throughout the cerebellar cortex express D2 receptors. Interestingly, deletion of the D2 receptors enhanced sociability and social recognition, whereas overexpression of D2 receptors resulted in impaired sociability and social recognition. Neither D2 receptor deletion nor overexpression influenced motor control as tested on the accelerating rotarod. These data were further recapitulated by D2 receptor deletion from solely Crus I and II (Fig. 1A-D), which, in line with other studies, suggests that Purkinje cells in Crus I and II are more important for the regulation of sociability than motor control [85, 92]. Nevertheless, the lack of motor abnormalities in both TH and D2 receptor knockout mice supports the hypothesis that the cerebellum regulates social and motor behaviors through different pathways.

**Table 1:** Effects of regional manipulations to the cerebellar cortex on motor and social behaviors. X = impairments; — = no impairments

Cell-type manipulated	Type of manipulation	Timing	Motor	Social
Inhibitory interneurons in Crus I/II	Chemogenetic activation	Juvenile	— [85]	X [85]
Purkinje cells in right Crus I	Chemogenetic inhibition	Adult	— [92]	X [92]
Purkinje cells in posterior vermis	Chemogenetic inhibition	Adult	— [42]	X [42]
Inhibitory interneurons in Crus I	Chemogenetic activation	Adult	X [85]	— [85]
Inhibitory interneurons in lobule VII	Chemogenetic inhibition	Adult	— [85]	X [85]
Inhibitory interneurons in lobule VII	Chemogenetic inhibition	Juvenile	— [85]	— [85]
Inhibitory interneurons in lobule VI/VII	Chemogenetic and optogenetic activation	Adult	— [86]	X [86]
Purkinje cells in lobule VII	Optogenetic inhibition	Adult	— [87]	X [87]
Inhibitory interneurons in lobule IV/V	Chemogenetic and optogenetic activation	Adult	— [86]	X [86]
All cell types in lobule IV/V	Lesion	Adult	X [88]	X [88]
Purkinje cells in lobule IV/V	Chemogenetic activation	Adult	X [88]	X [88]
Purkinje cells	*TH* deletion using *L7*^*Cre*^	Adult	— [90]	X [90]
Purkinje cells	*D2R* deletion or overexpression using viruses	Adult	— [91]	X [91]

### Cell Type-Specific Manipulations to Cerebellar Nuclei Neurons

In addition to the functional divergence of cerebellar input pathways, there is also evidence that anatomically and molecularly distinct cerebellar output pathways communicate with functionally distinct brain regions [29, 30, 53]. Therefore, specific subsets of cerebellar output pathways may contribute differently to social and motor behaviors (Table 2). Optogenetic inhibition of cerebellar nuclei axons in the ventral tegmental area causes impaired social preference without influencing motor performance on a parallel rod floor test [93]. In a second set of studies, researchers manipulated subsets of nuclei neurons by the selective deletion of genes encoding proteins (VGluT2 deletion) that are essential for loading neurotransmitters into synaptic vesicles, thereby effectively silencing these neurons [94]. Eliminating neurotransmission from a subset of glutamatergic nuclei neurons (*Ntsr1*^*+*^ neurons) resulted in impaired developmental motor reflexes without changes in vocalizations in a social isolation paradigm [61]. Interestingly, these same mice did not display any behavior deficits in adulthood. In contrast, adult mice lacking neurotransmission from all glutamatergic cerebellar nuclei neurons showed impaired motor performance on the rotarod without deficits in social preference [61]. Different contributions of glutamatergic and GABAergic nuclei neurons to motor and social behaviors were confirmed by a second paper. Here, researchers showed that chemogenetic inhibition of a subset of putative GABAergic neurons that express the dopamine receptor type 1 (D1R) in the dentate nucleus resulted in impaired social recognition without changes in motor performance on the accelerating rotarod [95]. Finally, chemogenetic inhibition of the dentate nucleus could reverse abnormal social behaviors caused by conditional deletion of an ASD gene from Purkinje cells, without rescuing motor abnormalities [42]. These data indicate that specific cerebellar nuclei projections can differentially control motor and social behaviors, that the timing and type of manipulation influence the behavioral outcome, and that glutamatergic and GABAergic neurons may differentially contribute to motor and social behaviors. These findings agree with the hypothesis that the cerebellum may mediate different behaviors through different cerebellar efferent pathways.

**Table 2:** Effects of nuclei cell-type specific manipulations on motor and social behaviors. X = impairments; — = no impairments

Cell-type manipulated	Type of manipulation	Timing	Motor	Social
VTA-projecting nuclei neurons	Optogenetic inhibition	Adult	— [93]	X [93]
*Ntsr1*^*+*^ nuclei neurons	*Vglut2* deletion / lack of neurotransmission	Early postnatal	X [61]	— [61]
*Ntsr1*^*+*^ nuclei neurons	*Vglut2* deletion / lack of neurotransmission	Adult	— [61]	— [61]
Glutamatergic nuclei neurons	*Vglut2* deletion / lack of neurotransmission	Adult	X [61]	— [61]
*D1R*^*+*^ nuclei neurons in dentate nucleus	Optogenetic inhibition	Adult	— [95]	X [95]

### Cerebellar Cortex-Wide Manipulations of Cellular, Synaptic, or Molecular Pathways

In contrast to regionally restricted or cell-type specific manipulations in the cerebellar cortex and nuclei, respectively, broad, cerebellum-wide manipulations to all Purkinje or granule cells (Fig. 1E) should affect all functional modules equally. In such studies, the cerebellum would be broadly manipulated in a cortex-wide manner and broadly affect the cellular, synaptic, or genetic pathways throughout the cerebellum. Because these manipulations exhibit no regional specificity and therefore should equally impair cerebellar regions involved in all cerebellar-dependent behaviors. If the cerebellum contributes to these behaviors via identical cellular, synaptic, or genetic pathways within each functional domain, then these manipulations should equally impair (or preserve) social and motor behaviors (Table 3).

### Manipulations to Granule Cell Proliferation

A few papers have investigated the contribution of granule cell (Fig. 1E) proliferation and integration into the cerebellar cortex on behaviors. Granule cells are the latest-born neurons in the cerebellum, and developmental perturbations to proliferating granule cells may underlie behavioral deficits in prematurely born infants [13, 96–99]. Prohibiting the neurogenesis of glutamatergic granule cells in the cerebellar cortex and glutamatergic nuclei results in small, infoliated cerebellums. In these mice, developmental motor reflexes are impaired, and fewer vocalizations in a social isolation paradigm are observed [100]. Using a different experimental manipulation, researchers caused excessive granule cell neurogenesis, which resulted in enlarged cerebellums [101], reduced motor control on the accelerating rotarod [101], and caused impairments in social preference and social novelty tests [102]. Finally, deleting an ASD-related gene that is important for neurogenesis from granule cell precursors, *Chd8*, also reduced granule cell proliferation and caused cerebellar hypoplasia throughout the cerebellar cortex. One group reported that rotarod performance, social preference, and social recognition were all impaired in these mice [103], but a second group did not replicate these findings of social deficits in mice with a conditional deletion of *Chd8* [104]. The disparity between these results may be caused by slight differences in the genetic constructs used to generate the two mouse lines used by these research groups. Thus, studies from three mouse models support the hypothesis that cerebellar cortex-wide changes to the granule cell neurogenesis in the cerebellum result in both social and motor behaviors, whereas one study found abnormalities in motor but not social behaviors.

### Manipulations to Synaptic Transmission between Specific Cells in Cerebellar Microcircuits

In addition to studies manipulating the cellular make-up of the cerebellar cortex, other studies have investigated how changing the synaptic function between specific cerebellar cell types across the cerebellar cortex influences different behaviors. These studies were inspired by the importance of many specific synapses and synaptic plasticity in motor control and motor learning. Genetic elimination of neurotransmission from climbing fibers [105] or parallel fibers [61] (Fig. 1E) resulted in fewer vocalizations upon social isolation and severely impaired developmental motor reflexes in early postnatal mice. These studies confirm that both major sources of glutamatergic inputs onto Purkinje cells (Fig. 1E) are important for both social and motor behaviors.

Reduced GABAergic transmission onto granule cells resulted in impaired social novelty and maternal behaviors in female mice but no social deficits in male mice. This manipulation also did not affect motor performance on the accelerating rotarod in either female or male mice [106]. Conversely, six mouse models with deficits in motor control or motor learning [107–112] did not have deficits in social preference or social recognition tests [111–113]. These included mice with the majority of granule cells silenced (impaired motor performance on accelerating and impaired vestibular-ocular reflex) [107], mice with impaired synaptic inhibition onto Purkinje cells (impaired vestibular-ocular reflex) [109], two mouse models wherein parallel fiber to Purkinje cell synaptic plasticity was impaired (impaired vestibular-ocular reflex) [108, 110], and two mouse models with impaired trafficking of glutamate receptors (mGlu1) in Purkinje cells (impaired motor performance on accelerating rotarod) [111, 112]. These findings show that motor and social behaviors can be differentially affected when different synapses in the cerebellar circuit are manipulated (Fig. 1E). These data challenge the hypothesis that all cerebellar synapses are equally engaged in all cerebellar-dependent behaviors.

### Deleting Disease-Associated Genes Across the Cerebellar Cortex

In addition to investigating the effects of cellular composition in the cerebellar circuitry or function of specific synapses, many studies have used the conditional deletion of genes in cerebellar Purkinje cells or granule cells (Fig. 1E) to investigate how these genes contribute to social and motor behaviors. In the first of such studies, Tsai and colleagues showed that Purkinje cell-specific deletion of the ASD-associated gene *Tsc1* resulted in reduced social preference and impaired performance in the social recognition test, and these *Tsc1* conditional knockout mice also showed abnormal gait [19]. Since then, the importance of ASD-associated genes for Purkinje cell function and social and motor behaviors has been confirmed using multiple conditional knockout mice. These include Purkinje cell-specific conditional deletion of *PTEN* (reduced social preference and reduced rotarod performance) [114], *Tsc2f* (reduced social preference and reduced rotarod performance) [115], *Shank2* (reduced social preference, reduced motor performance on the Erasmus Ladder, and impaired vestibular-ocular reflex in one study [116]; no social impairments but reduced motor performance on the Erasmus Ladder in another study [117]), and *Scna8* (reduced social preference and social recognition, reduced performance on the rotarod, and gait abnormalities) [118]. Additionally, cerebellum-wide conditional deletion of *Auts2* resulted in impaired motor performance on the rotarod and fewer male courtship vocalizations [119]. These papers all provide evidence in support of the theory that social and motor behaviors rely on similar molecular pathways in the cerebellum.

*Tsc1, PTEN*, and *Tsc2f* all encode proteins in the mTOR pathway, leading researchers to further dive into the contribution of the mTOR pathway to cerebellar control of social and motor behaviors. *Tsc1* Purkinje cell conditional knockout mice were treated with rapamycin to test whether normalizing mTOR function could rescue social and motor behaviors [120]. Early rapamycin treatment normalized social and motor behaviors, but late treatment only rescued motor impairment. Treatment success for social behaviors, but not motor control, relied on Purkinje cell survival in addition to rapamycin treatment. Thus, at the molecular level, social and motor behaviors are differentially dependent on mTOR signaling and Purkinje cell survival. This was further underscored by studies showing that mTORC1 signaling is essential for motor but not social behaviors [121–123], whereas mTORC2 signaling is essential for social but not motor behaviors [121].

Deletion of other genes also shows the differential importance of certain molecular pathways for social and motor behaviors. For example, *MeCP2* deletion from the entire cerebellum resulted in impaired performance on the rotarod but no abnormal social preference [124], although *MeCP2* deletion from Purkinje cells specifically led to abnormal motor performance on the accelerating rotarod, impaired vestibular ocular reflexes, and reduced social recognition [125]. In contrast, deletion of the schizophrenia-associated gene *DISC* from Purkinje cells resulted in reduced social recognition without affecting motor performance on the rotarod [126]. Similarly, deletion of the fragile X syndrome gene *Fmr1* from Purkinje cells resulted in reduced social preference and recognition without affecting performance on the rotarod, and re-expression of *Fmr1* solely in Purkinje cells largely rescued these social deficits [127]. Together, these findings suggest that the molecular pathways required for cerebellum-mediated social and motor behaviors are not identical.

In summary (Table 3), while there are some mouse models with cerebellum-specific and cerebellum-wide manipulations that show impairments in both motor and social behaviors [19, 61, 100–102, 105, 115, 118, 119, 125], there are also many mouse models that show differential reliance on certain cellular, synaptic, aminergic, and molecular pathways between motor and social behaviors [90, 91, 106, 113, 120–124, 126, 127]. These findings do not support the hypothesis that the cerebellum contributes to all behavioral domains through identical cellular, synaptic, and molecular pathways.

**Table 3:** Effects of cortex-wide manipulations on motor and social behaviors. X = impairments; — = no impairments; ♀ = female; ♂ = male. *Atoh1*^*Cre*^ is expressed in glutamatergic cerebellar neurons during development; *En1*^*Cre*^ is expressed in all cerebellar neurons during development; *Gabra6*^*Cre*^ is expressed in mature pontine neurons and granule cells; *L7*^*Cre*^ is expressed in all mature Purkinje cells; *Ptf1a*^*Cre*^ is expressed in GABAergic cerebellar neurons and glutamatergic inferior olive neurons during development

Cell-type manipulated	Type of manipulation	Timing	Motor	Social
Granule cerebellar neurons	*Atoh1* deletion using *En1*^*Cre*^ / no neurogenesis	Early postnatal	X [100]	X [100]
Granule cells	*Ngfr* deletion using *Atoh1*^*Cre*^ / excessive proliferation	Adult	X [101]	X [102]
Granule cells	*Chd8* deletion using *Atoh1*^*Cre*^ / diminished proliferation	Adult	X / X [103] / [104]	X / — [103] / [104]
Granule cells	*Vglut2* deletion using *Atoh1*^*Cre*^ / lack of neurotransmission	Early postnatal	X [61]	X [61]
Inferior olive neurons	*Vglut2* deletion using *Ptf1a*^*Cre*^ / lack of neurotransmission	Early postnatal	X [105]	X [105]
Granule cells	*δGABA*_*A*_ deletion using *Gabra6*^*Cre*^ / granule cell hyperexcitability	Adult	— [106]	♀: X / ♂: — [106]
Granule cells	*CaV2.1* deletion using *Gabra6*^*Cre*^ / granule cell silencing	Adult	X [107]	— [113]
Purkinje cells	*Gabrg2* deletion using *L7*^*Cre*^ / lack of synaptic inhibition	Adult	X [109]	— [113]
Purkinje cells	*PKCI* knock-in in *L7* locus / LTD blockade	Adult	X [108]	— [113]
Purkinje cells	*PP2B* deletion using *L7*^*Cre*^ / LTP blockade	Adult	X [110]	— [113]
Purkinje cells	*KIF2C* deletion using *L7*^*Cre*^ / impaired mGlu1 trafficking / abnormal parallel fiber input	Adult	X [111]	— [111]
Purkinje cells	*TFR1* deletion using *L7*^*Cre*^ / impaired mGlu1 trafficking / abnormal parallel fiber input	Adult	X [112]	— [112]
Purkinje cells	*Tsc1* deletion using *L7*^*Cre*^	Adult	X [19]	X [19]
Purkinje cells	*PTEN* deletion using *L7*^*Cre*^	Adult	X [114]	X [114]
Purkinje cells	*Tsc2f* deletion using *L7*^*Cre*^	Adult	X [115]	X [115]
Purkinje cells	*Shank2* deletion using *L7*^*Cre*^	Adult	X / X [116] / [117]	X / — [116] / [117]
Purkinje cells	*Scna8* deletion using *L7*^*Cre*^	Adult	X [118]	X [118]
All cerebellar neurons	*Auts2* deletion using *En1*^*Cre*^	Adult	X [119]	♂: X [119]
Purkinje cells	*Tsc1* deletion using *L7*^*Cre*^ + early rapamycin treatment	Adult	— [120]	— [120]
Purkinje cells	*Tsc1* deletion using *L7*^*Cre*^ + late rapamycin treatment	Adult	— [120]	X [120]
Purkinje cells	*Rptor* deletion using *L7*^*Cre*^	Adult	X [121–123]	— [121–123]
Purkinje cells	*Rictor* deletion using *L7*^*Cre*^	Adult	— [121]	X [121]
All cerebellar neurons	*MeCP2* deletion using *En1*^*Cre*^	Adult	X [124]	— [124]
Purkinje cells	*MeCP2* deletion using *L7*^*Cre*^	Adult	X [125]	X [125]
Purkinje cells	*DISC* deletion using *L7*^*Cre*^	Adult	— [126]	X [126]
Purkinje cells	*Fmr1* deletion using *L7*^*Cre*^	Adult	— [127]	X [127]

## Discussion

This review summarizes results from cerebellum-specific chemo-, opto-, and conditional genetic mouse models that were tested on at least one behavioral task in the social domain and one behavioral task in the motor domain. The first of these studies was published only a little over a decade ago [19] but now dozens of papers have investigated the cerebellum’s role in social and motor behaviors using cerebellum-specific manipulations. The findings summarized in this review confirm the importance of the cerebellum for normal social behaviors and suggest that cerebellar dysfunction directly contributes to myriad symptoms in patients. Having answered the question of whether the cerebellum is important for social behaviors, the next logical problem to address is whether the cerebellum affects social and other cognitive functions via the same mechanisms through which it affects other behaviors, like motor control. The research presented in this review article suggests that the mechanism by which the cerebellum contributes to diverse behaviors is more complex than through identical pathways.

The specific behavioral tasks used to assess social and motor functions are not identical between all studies described in this literature review, and even for similar behavioral paradigms, there can be slight differences in the behavioral protocols between different research teams. This limits the ability to make direct comparisons between all research findings described in this review. Nevertheless, regardless of the precise behavioral tasks used to assess social and motor domains, many studies showed impairment in one domain (i.e. a social behavior task) without impairments in the other domain (i.e. a motor behavior task) within the *same* cerebellum-specific opto-, chemo-, or conditional genetic mouse model. These findings confirm that not all cerebellum-dependent behaviors rely on the same neural pathways or genes.

The lack of convergence on identical cellular, synaptic, and molecular pathways essential for social and motor behaviors does not have to contradict the hypothesis of similar neural computations. For example, a range of gene expression and synaptic weights can lead to the same circuit behavior in the crab stomatogastric ganglion [128–131]. Yet, unlike in the crab stomatogastric ganglion, how cerebellar activity translates to specific behaviors is unknown. Evidence suggests that specific cerebellar spike train signatures relate to distinct patterns of motor impairments [132], yet it is unknown whether there are also unique neural signatures for impairment in different types of social behaviors, such as social preference, social recognition, social vocalizations, and maternal behaviors. Understanding whether cerebellar neural activity, specifically Purkinje cell spike patterns, is the same across cerebellar regions for different task domains would require in vivo recordings from different parts of the cerebellum and across behavioral states. These types of experiments would provide insight into whether the neural activity of Purkinje cells converges on similar patterns for different behavioral domains, despite diverging employment of distinct cellular, synaptic, or molecular pathways.

Even if dissimilarities are observed in Purkinje cell activity across behavioral domains, the effect of this activity on other brain regions may be similar. Recent work has suggested that the cerebellum may cause other brain regions to function on the same wavelength and thereby optimize the ability of diverse brain regions to work together on a common task [36, 133–137]. This theoretical framework allows for differences in computational processes across functional and anatomical cerebellar domains, with commonality in the *outcome* of these computations being task-specific collaborations between specialized cerebral regions. Dissimilar neural activity within the cerebellum may be necessary to increase collaborations and synchronization of wavelengths between specialized cerebral cortices because these cerebral cortices each have unique neural properties. The need for dissimilar neural activity could explain why the cellular, synaptic, and molecular pathways necessary for social and motor tasks are not always identical. Testing whether the cerebellum orchestrates collaboration across brain regions would require neural recordings across the cerebral cortex and behavioral states in mice with and without cerebellar dysfunction. If true, there may be many underlying pathways and activity patterns in the cerebellum for distinct behavioral tasks that are yet to be uncovered. However, this would allow researchers to describe the cerebellum’s singular neural function as orchestrating wavelengths across specialized brain regions for context-specific neural collaboration on complex tasks.

Altogether, experimental mouse studies have provided undeniable proof that normal cerebellar function is necessary for behavioral domains beyond motor control, including social behaviors. These studies are supported by anatomical studies that show that the cerebellum has direct and indirect connections throughout the brain. However, anatomical studies cannot fully explain how the cerebellar cortex integrates and modulates information about specific behaviors. The results reported in this review suggest that different cellular, synaptic, and molecular pathways are employed for different behavioral domains. Now that the question of *whether* the cerebellum contributes to behaviors beyond motor control is settled, the question of *how* the cerebellum contributes to many distinct behaviors remains largely elusive. Answering this question may explain why patients with cerebellar dysfunction may present with a spectrum of symptoms that range in severities across different neural domains.

## Data Availability

No datasets were generated or analysed during the current study.
